# Hybrid Nanocomposite Mini-Tablet to Be Applied into the Post-Extraction Socket: Matching the Potentialities of Resveratrol-Loaded Lipid Nanoparticles and Hydroxyapatite to Promote Alveolar Wound Healing

**DOI:** 10.3390/pharmaceutics17010112

**Published:** 2025-01-15

**Authors:** Viviana De Caro, Giada Tranchida, Cecilia La Mantia, Bartolomeo Megna, Giuseppe Angellotti, Giulia Di Prima

**Affiliations:** 1 Dipartimento di Scienze e Tecnologie Biologiche Chimiche e Farmaceutiche (STEBICEF), University of Palermo, Via Archirafi 32, 90123 Palermo, Italy; viviana.decaro@unipa.it (V.D.C.); cecilialamantia000@gmail.com (C.L.M.); 2 Dipartimento di Ingegneria, University of Palermo, Viale delle Scienze, 90128 Palermo, Italy; giada.tranchida@unipa.it (G.T.); bartolomeo.megna@unipa.it (B.M.); 3 Istituto per lo Studio dei Materiali Nanostrutturati, Consiglio Nazionale delle Ricerche (ISMN-CNR), Via Ugo La Malfa 153, 90146 Palermo, Italy; giuseppe.angellotti@cnr.it

**Keywords:** nanocomposite, resveratrol, 18-β-glycyrrhetinic acid, lipid nanoparticles, hydroxyapatite, post-extraction socket, wound healing, mini-tablet, ex vivo

## Abstract

**Background/Objectives**: Following tooth extraction, resveratrol (RSV) can support healing by reducing inflammation and microbial risks, though its poor solubility limits its effectiveness. This study aims to develop a solid nanocomposite by embedding RSV in lipid nanoparticles (mLNP) within a hydrophilic matrix, to the scope of improving local delivery and enhancing healing. Hydroxyapatite (HXA), often used as a bone substitute, was added to prevent post-extraction alveolus volume reduction. **Methods**: The mLNP-RSV dispersion was mixed with seven different polymers in various mLNP/polymer ratios. Following freeze-drying, the powders were redispersed, and the resulting dispersions were tested by DLS experiments. Then, the best two nanocomposites underwent extensive characterization by SEM, XRD, FTIR, Raman spectroscopy, and thermal analysis as well as in vitro partitioning studies aimed at verifying their ability to yield the mLNP-RSV from the hydrophilic matrix to a lipophilic tissue. The characterizations led to identify the best nanocomposite, which was further combined with HXA to obtain hybrid nanocomposites, further evaluated as pharmaceutical powders or in form of mini-tablets. **Results**: PEG-based nanocomposites emerged as optimal and, following HXA insertion, the resulting powders revealed adequate bulk properties, making them useful as a pharmaceutical intermediate to produce ≈59 mm^3^ mini-tablets, compliant with the post-extraction socket. Moreover, they were proven ex vivo to be able to promote RSV and GA accumulation into the buccal tissue over time. **Conclusions**: The here-proposed mini-tablet offers an innovative therapeutic approach for alveolar wound healing promotion as they led to a standardized dose administration, while being handy and stable in terms of physical solid identity as long as it takes to suture the wound.

## 1. Introduction

Tooth extraction is one of the most common surgical procedures in dentistry [1]. The healing process of the socket involves both soft (e.g., periodontal ligament, gum) and hard tissues (e.g., alveolar bone) [2]. The healing of the socket after tooth extraction occurs in four distinct phases. The first one is governed by hemostasis and coagulation; bleeding from the socket allows platelets to interact with endothelial cells and the extracellular matrix, starting the coagulation cascade that forms an initial blood clot, establishing hemostasis and creating a structure for further cell adhesion [3]. In the second phase, cytokines and growth factors play their role in the inflammatory stage, leading to the organization of the fibrin clot, which is replaced by granulation tissue. In the third phase there is rapid deposition of a temporary matrix (fibroplasia) which displaces granulation tissue and periodontal ligament remnants, followed by the formation of woven bone around newly developed blood vessels. Finally, the modeling and remodeling stages reshape the bone structure involving the deposition of lamellar and bone marrow, replacing woven bone without affecting overall bone shape or architecture. Osteoblasts and osteoclasts are crucial in this phase, during which both bone and surrounding soft tissue loss may be observed due to increased osteoclastic activity [1,4,5,6]. The exacerbation and prolongation of even just one of these phases leads to an incomplete healing process. Since the four phases are all interconnected, and considering the ability of inflammatory processes to self-sustain, as well as the importance of reactive oxygen species (ROS) in the various signaling pathways involved in the wound repair process, a strategy to ensure prompt healing includes the use of natural substances with scavenging, anti-inflammatory and antimicrobial activities. Nowadays, innovative dental materials, intended as mouthwashes, endodontics, orthodontics and filling materials, containing natural phyto-components are an emerging trend due to their efficacy, low cost, and non-toxicity against humans. Particularly, polyphenols belong to an emerging class of useful natural bioactives in dentistry [7]. Among them, resveratrol (RSV) currently stands out. It is a polyphenolic phytoalexin, belonging to the stilbene subfamily, capable of exerting antioxidant, anti-inflammatory, and cardioprotective actions [8,9,10]. RSV exerts protective effects on bone tissue and, also, bone formation, since it is an active against osteoporosis and bone resorption due to aging [11,12]; as well as it can also stimulate the proliferation and differentiation of osteoblasts [13]. Furthermore, it possesses antimicrobial activity which could be helpful in protecting the damaged tissue from microbial colonization that otherwise could slow down or prevent the healing process [14]. Unfortunately, RSV has an unfavorable pharmacokinetic profile due to its low solubility in aqueous media and high susceptibility to the hepatic first-pass metabolism, resulting in a bioavailability of less than 1% when taken orally [15]. Consequently, considering that the post-extraction socket healing process is limited to a certain area of the oral cavity, it should be more effective to act locally. Nevertheless, RSV is unwieldy due to the already mentioned poor water solubility together with instability due to light exposure, alkaline pH, and high temperatures [16]. Therefore, considering its lipophilic nature, its insertion into lipid-based nano-platforms could represent a valuable solution to protect RSV and promote its interaction with soft tissues which also have hydrophobic nature. Lipid-based nano-platforms have evolved over the years: (i) the Solid Lipid Nanoparticles (SLN) are composed of physiologically tolerated solid lipids, finely dispersed in an aqueous phase containing at least one surfactant [17]; (ii) the Nanostructured Lipid Carriers (NLC) consist of a mixture of at least two lipids, one solid and one liquid at room temperature and pressure, allowing higher stability and drug encapsulation capacity than the SLN [18]; (iii) the Multicomponent Lipid Nanoparticles (mLNP) represent a further progression of the NLC as they are still based on a mixture of at least two lipids (one solid and one liquid) but also present some functional components capable of assisting and synergizing the therapeutic effect of the encapsulated active ingredient, e.g., for wound healing purposes, functional excipients characterized by proper scavenging and antimicrobial properties should be chosen. Specifically, we have already developed and characterized RSV-loaded mLNPs based on a mixture of Labrasol^®^ (liquid lipid), Glyceryl Monostearate (solid lipid), Menthol, and 18-β-Glycyrrhetinic Acid (functional lipids) [19]. These functional excipients were generally chosen for their wound healing potential. Specifically, glycyrrhetinic acid (GA) is a natural triterpene glycoconjugate present in licorice roots and it is a well-known HMGB1 inhibitor. GA has been proven to possess immunomodulatory, antioxidant, antibacterial, and anti-inflammatory activities, and has been widely used to clinically treat such chronic inflammatory diseases (e.g., chronic hepatitis, atopic dermatitis and other skin diseases) [20,21]. Furthermore, a recent study published in 2018 underlined its osteoprotective ability by inhibiting osteoclasts activity [22]. On the other hand, menthol is a natural terpene that has been widely investigated as a penetration enhancer as well as for its antimicrobial potential, enabling both the maintenance of wound asepsis and the promotion of RSV entry into the target injured tissue [23,24]. The accurate design of the proposed mLNP-RSV led to a promising nanosystem which displayed powerful antioxidant activity due to the synergistic effect of RSV and GA, as highlighted by the DPPH assay, as well as interesting wound healing properties, as proven by a scratch assay on fibroblasts [19]

Considering that together with persisting inflammation and microbial colonization, a further issue to be addressed in post-extraction socket healing is related to the reduction in alveolus volume, which physiologically occurs following extraction; the objective of this work was to propose a therapeutic system capable of supporting alveolar healing in a comprehensive manner. Generally, to minimize the phenomenon of alveolar volume reduction, it is possible to use a bone substitute such as hydroxyapatite (HXA; Ca_10_(PO_4_)_6_(OH)_2_). At present, HXA is the mainly used bone substitute as it is able to create a close bond with the bone tissue, while also promoting osteoconduction. Moreover, it is easily bioabsorbable and has no negative effects on the organism [25].

Therefore, the final aim of this work is to create a hybrid solid nanocomposite, containing HXA and the already characterized mLNP loaded with RSV, both being dispersed into a hydrophilic matrix, which could be directly inserted inside the post-extraction socket to promote the wound healing process.

## 2. Materials and Methods

### 2.1. Materials

Trans-Resveratrol (RSV) and 18-β-Glycyrrhetinic Acid (GA) were purchased from A.C.E.F. spa (Fiorenzuola D’Arda, Piacenza, Italy). Labrasol^®^ (LB) was kindly supplied by Gattefossé (Lyon, France). Glyceryl Monostearate 55–60 (GMS) was obtained from Farmalabor (Canosa di Puglia, Italy). Menthol (ME), Polyethylene glycol 6000 (PEG6K), Polyethylene glycol 10000 (PEG10K), Hyaluronic acid sodium salt (HA), Carboxymethylcellulose (CMC), Polyvinylalcohol (PVA), Polyvinylpyrrolidone K90 (PVP K90), and Polyvinylpyrrolidone K30 (PVP K30) were purchased from Carlo Erba Reagents (Milan, Italy). The Hydroxyapatite (HXA) was purchased as SpherHA granules (dense granules, 0.5–1 mm, SHA-D1006) from Tiss’You srl (Domagnano, Republic of San Marino). β-cyclodextrin (β-CD) was obtained from Roquette Italia S.P.A (Cassano Spinola, AL, Italy). Pluronic F-127 was supplied by Sigma Aldrich (Milan, Italy). Trifluoroacetic acid (TFA) was obtained from Merck (Darmstadt, Germany). Sodium Citrate Dihydrate and Citric Acid Monohydrate were supplied by VWR International (Leuven, Belgium). The citrate buffer pH 5.5 was prepared by dissolving 3.024 g of sodium citrate dihydrate and 0.636 g of Citric Acid Monohydrate in 1 L of ultrapure water.

All chemicals and solvents (analytical grade) were purchased from Carlo Erba Reagents (Milan, Italy) and were used without any further purification.

### 2.2. Preparation and Characterization of the mLNP-RSV

The RSV-loaded multicomponent lipid nanoparticles (mLNP-RSV) were obtained and characterized as previously described [19]. Firstly, an RSV-loaded lipid mixture consisting of LB (30% *w*/*w*), GMS (60% *w*/*w*), GA (3% *w*/*w*), ME (2% *w*/*w*), and RSV (5% *w*/*w*) was prepared 3 times and subjected to quantitative analyses (HPLC-DAD, see below), which were carried out in triplicate. Results are reported as means (n = 9) ± standard error (SE).

**Scheme 1:**
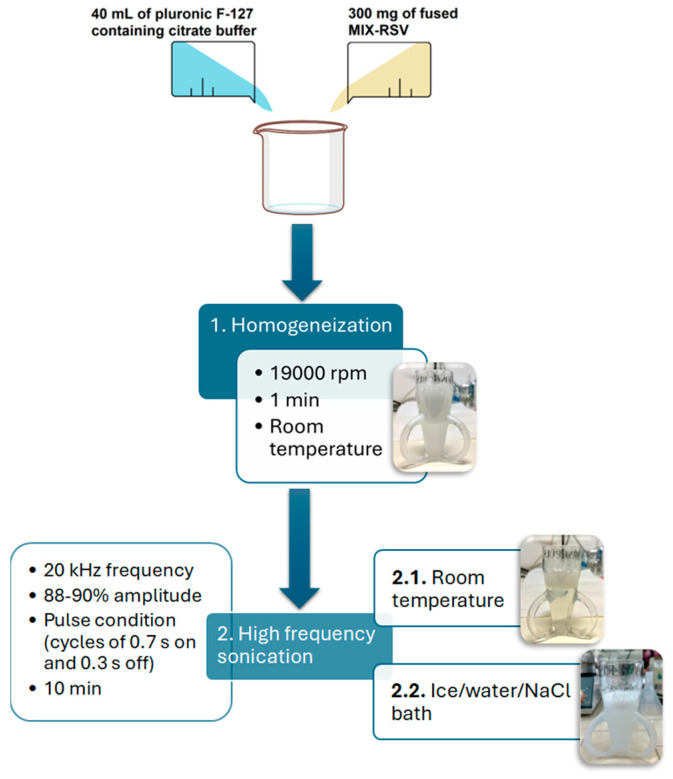
Preparation of the mLNP-RSV [19].

The characterized lipid mixture was then used to prepare the mLNP-RSV using the hot melt technique as previously reported and according to Scheme 1. The resulting nanoparticles were characterized in terms of RSV and GA contents (expressed as Drug Recovery %, Drug Loading % and Loading Efficacy %), particle size, PDI, and Z-potential, in accordance with the already published methods, in order to confirm sample characteristics. Results are reported as means (n = 9) ± SE.

### 2.3. Polymer Selection and Preparation of the Nanocomposites

Seven different natural polymers were tested to obtain the nanocomposite. HA, CMC, PVA, PVP K30, PVP K90, PEG6K, and PEG10K dispersion in HPLC-grade water were prepared at various concentrations. Then, 750 μL of the latter dispersions were mixed with 750 μL of fresh mLNP-RSV dispersion in order to obtain the desired weight ratios between mLNP-RSV and the polymer (from a minimum of 1:0.5 to a maximum of 1:8). Therefore, the dispersions were stored at −80 °C overnight (Thermo Forma ultra-freezer −86 °C. mod.902, Thermo Scientific, Waltham, MA, USA), and then subjected to freeze-drying for 72 h at 0.014 mPa and ≈−50 °C (Labconco FreeZone^®^ 2.5 Liter Freeze Dry System, Kansas City, MO, USA). Each weight ratio was tested in triplicate (n = 3).

### 2.4. Redisperdisility, DLS, and Z-Potential of the mLNP-RSV Embedded into the Nanocomposites

Following the freeze-drying process, the obtained solid nanocomposites were reconstituted with 750 μL of HPLC-grade water, with the aid of a vortex (LLG-uni TEXER 1). Then, the redispersed samples were subjected to a visual investigation aimed at evaluating the aspect of the dispersion and the time required for the redispersion process. Additionally, samples were diluted 1:99 with HPLC-grade water and subjected to Dynamic Light Scattering (DLS) studies at 25.0 ± 0.5 °C using a Malvern Zetasizer NanoZS instrument (Malvern, Worcestershire, UK) (λ_laser_ = 532 nm; fixed scattering angle = 173°). The particles’ diameter and polydispersity index (PDI), obtained through cumulative analyses of the correlation function, were considered. Furthermore, the Z-potential was also evaluated [26].

The experiments were performed in triplicate and results are reported as means (n = 3) ± SE. Only the mLNP-P6K (F) and mLNP-P10K (F) samples were selected for further studies and indicated as mLNP-RSV-P6K and mLNP-RSV-P10K.

### 2.5. Scanning Electron Microscopy

The morphology of the mLNP-RSV after their loading into the nanocomposites was observed by Scanning Electron Microscopy (SEM) consisting of a Philips Quanta 200 ESEM microscope (Philips, Amsterdam, The Netherlands) coupled with energy dispersive X-ray (EDX) analysis facilities. Pure solid RSV was transferred on a double-sided adhesive carbon tape and then photographed at different magnification in high vacuum condition at a voltage of 10 kV. Conversely, the solid nanocomposites were re-dispersed in deionized water at different dilutions. After slight agitation, some drops were placed on a metal plate and then dried. Pictures were taken after gold spraying at 30 mA for 30 s using an ion sputter coater (HHV Scancoat Six, Edwards Ltd., Burgess Hill, UK) at a pressure of 8–10^−1^ Pa. Images were taken at different magnifications in high vacuum condition at 20 kV.

### 2.6. X-ray Diffraction

The structure of the nanocomposites was investigated through X-ray Diffraction (XRD), involving the use of an X-ray diffractometer (Malvern Panalytical Empyrean, Malvern, UK) equipped with a Cu anode with Kα radiation (λ = 0.15405 nm) and PixCel^®^ 1D detecter. The operating voltage and current were 40 kV and 40 mA, respectively. XRD patterns were obtained using a theta-theta configuration with a scan range of 10–90 degrees. Powdered samples were pressed on a glass microscope slide, allowing a background free signal.

### 2.7. Fourier-Transform Infrared Spectroscopy

FTIR spectroscopy was performed using a Shimadzu FTIR 8000 (Kyoto, Japan) in the wavenumber range of 400–4000 cm^−1^ with a 4 cm^−1^ spectral resolution. For transmission, a KBr pellet with an approximate 2:100 weight ratio of sample and KBr was used for the preparation of the samples.

### 2.8. Raman Spectroscopy

A Renishaw Invia (Wotton-under-Edge, UK) Raman spectrometer coupled with a Leica MSDS Microscope (50× LWD lens) and a CCD detector was used for the acquisition of Raman spectra. A solid-state laser (Nd:YAG) at 532 nm was used for the excitation. The power of the incident beam was 5 mW while the width of the analyzed spot was about 2 μm. The Raman analysis was performed at different points of each sample in order to assess the reliability of the measurement.

### 2.9. Thermal Analysis

Simultaneous Thermal Analysis was conducted in a Netzsch Jupiter F1 STA 449 (NETZSCH B.V & Co.Holding KG, Selb, Germany) in the range 30–1000 °C with a heating rate of 10 °C min^−1^ by fluxing 20 mL min^−1^ of nitrogen and 20 mL min^−1^ of air during the analysis.

### 2.10. In Vitro Partitioning Studies of the mLNP-RSV from a Hydrophilic to a Lipophilic Compartment

The ability of the mLNP-RSV to partition from a hydrophilic compartment, i.e., the environment in which they are dispersed, to a lipophilic one, i.e., 1-octanol, was evaluated using Transwell-type polycarbonate inserts (MultiScreen^®^ Filter Plate, Millipore, Sigma Aldrich, Milan, Italy) with a cut-off equal to 0.45 µm. A measure of 200 μL of mLNP-RSV dispersion, both the fresh one or the ones reconstituted after freeze-drying, were placed inside the Transwell insert, which was further placed inside a beaker pre-filled with 10 mL of 1-octanol [27]. This system was maintained at room temperature under constant magnetic stirring (200 rpm) for 28 h. At pre-established time intervals, 1 mL aliquots of the acceptor medium were withdrawn and immediately replaced with 1 mL of fresh 1-octanol to maintain the sink conditions. These samples were diluted with methanol (1:4 *v*/*v*) and then subjected to UV-Vis analyses (Shimadzu 1700 instrument, Kyoto, Japan) for RSV quantification. For this reason, a calibration curve was constructed by analyzing 5 standard solutions of RSV in methanol:

- λ_max_ = 305 nm; linearity range: 0.1–5.0 μg/mL; linear regression: y = 129.64 x + 0.00264 [mg/mL] (R = 0.999).

The partition of free RSV as a solution was also evaluated under the same experimental conditions. Therefore, 200 μL of an aqueous RSV solution, having the same concentration as the RSV in the analyzed mLNP-RSV dispersions, was loaded into the Transwell. To this aim, 1 mg/mL RSV stock solution in citrate buffer pH 5.5 + PEG200 (4:1 *v*/*v*) was prepared and then diluted with citrate buffer pH 5.5 to achieve the desired concentration.

All experiments were performed in triplicate (n = 3) and the results are expressed as the RSV dose fraction as a function of time (means ± SE). Further experiments were conducted only with the mLNP-P10K nanocomposite.

### 2.11. Preparation and Characterization of Hybrid Nanocomposites Containing Hydroxyapatite and mLNP-RSV (mLNP-RSV-P10K-HXA)

To include the hydroxyapatite (HXA) into the chosen nanocomposite, two different approaches were tested. The first approach consists of producing a physical mixture of the appropriate amount of mLNP-P10K powder with the grounded HXA with the aid of a vortex (mLNP-RSV-P10K-HXA-1). The second approach consisted of including the HXA before the freeze-drying process of mLNP together with PEG10K. In this case, the desired amount of HXA was added to the PEG10K dispersion and then the resulting milky suspension was vortexed, mixed with the freshly prepared mLNP-RSV dispersion, immediately frozen in liquid nitrogen, and freeze-dried as previously described (mLNP-RSV-P10K-HXA-2). In both cases the following PEG10K:HXA weight ratios were tested: 1:0.25; 1:0.5; 1:1; 1:1.25; 1:1.5 (*w*/*w*).

The mLNP-P10K-HXA hybrid nanocomposites were immediately subjected to a visual redispersion assessment. Each HXA-including nanocomposite was prepared 3 times.

The amount of RSV and GA loaded into the resulting biomaterial was assessed by HPLC-DAD analyses as reported below. Briefly, an accurately weighted amount of powder was dissolved in methanol, centrifuged, filtered, and analyzed. Results are expressed as Drug Loading % (*DL*%):

$$DL\%=\frac{loaded\;amount\;of\;RSV\;or\;GA\;\left(\mathrm{mg}\right)}{weighted\;nanocomposite\;\left(\mathrm{mg}\right)}\times 100$$

Quantitative analyses were repeated three times on each prepared nanocomposite and the results are expressed as means (n = 9) ± SE.

Moreover, the morphology of the nanocomposites was observed by optical microscopy using a Reichert-Jung Microstar 110 optical microscope (Reichert, Buffalo, NY, USA) to assess the geometric characteristics of powder. The images were captured with an external camera at 4× and 10× magnification.

### 2.12. Bulk Properties of the Nanocomposite Powders

The bulk and tapped volumes of both hybrid and not-hybrid nanocomposite powders were determined according to *FUI XII ed.* by pouring an accurately weighed amount of powder into a graduated cylinder. The bulk volume (V_B_) was immediately recorded. Subsequently, the cylinder was subjected to a series of taps (10, 500, and 1250 taps) to promote the packing of the powder mass and reduce the interparticle spaces between them. After the tapping process was completed, the tapped volume (V_T_) was measured. The analyses were conducted in triplicate using a single preparation batch and were used to mathematically calculate the bulk (ρ_B_) and tapped densities (ρ_T_) by relating the mass of the nanocomposite to its volumes. The results are reported as means (n = 3) ± SE. The results obtained were then used to predict the flow characteristics of the powder by calculating the Hausner ratio (*H*) and the Carr index (or Compressibility index %) according to the following equations reported in *FUI XII ed.*:

$$H=\frac{\rho_{T}}{\rho_{B}}\;Carr\;Index=\frac{\left(\rho_{T}-\rho_{B}\right)}{\rho_{T}}\times 100$$

Furthermore, to experimentally assess the flow characteristics of the nanocomposite powders, the angle of repose was evaluated in accordance with *FU XII ed.* For this test, an INOX steel instrument (Flowability Tester Model BEP2, Copley Scientific, Nottingham, UK) was used, allowing the determination of the angle of repose. This instrument consists of a funnel with an adjustable diameter hole, below which a Petri dish with a diameter of 55 mm was placed. A measure of 500 mg of each nanocomposite was poured into the funnel and allowed to fall until the surface of the Petri dish was completely covered, forming a cone. The cone’s height was measured using a specific device (Mitutoyo Absolute Digimatic Heightgage, Copley Scientific, Nottingham, UK). The angle of repose of the powder (Φ) was then calculated using the following formula:

$$\Phi =\arctan\left(\frac{h}{r}\right)$$

where h is the height of the cone and r is the radius of the Petri dish. For the evaluation of the angle of repose of the nanocomposites, an orifice with a diameter of 15 mm was used. Each experiment was repeated 6 times, and the results are reported as mean (n = 6) ± SE.

### 2.13. Preparation of Mini-Tablets from Hybrid and Not-Hybrid Nanocomposite Powders

Randomly selected aliquots of each nanocomposite powder were carefully weighed and directly compressed (6 tons) using a hydraulic, single-die tableting machine (fixed diameter: 5 mm; Perkin Elmer IR Accessory, Waltham, MA, USA), two flat-faced punches, and a die [28]. To assess the reproducibility of the preparation method and the mini-tablets’ uniformity in terms of weight, thickness, and actives content, 6 tablets for each nanocomposite were evaluated using an analytical balance (Mettler Toledo AE240, Columbus, OH, USA), a digital micrometer (VWR International, Milan, Italy), and HPLC-DAD, as reported below. The results are expressed as means (n = 6) ± SE.

### 2.14. In Vitro Disintegration of the Hybrid and Not-Hybrid Nanocomposites: Powder vs. Mini-Tablet

To simulate the in vivo conditions (post-extraction socket) the disintegration time (DT) was evaluated by inserting a mini-tablet or an equal amount of nanocomposite powder into a glass crucible and adding 1 mL of citrate buffer solution (pH 5.5) prewarmed at 37.0 ± 0.5 °C [24]. During the experiment, multiple photographs and short videos were collected to visually assess the complete disintegration. The experiment was repeated in triplicate. The resulting DTs are reported as means (n = 3) ± SE.

### 2.15. Ex Vivo Evaluation of Penetration and/or Permeation Behavior of RSV and GA from Nanocomposites

#### 2.15.1. Tissue Preparation

Porcine buccal mucosa samples were obtained from the vestibular area of the retromolar trigone of 8–12-month-old domestic pigs. These specimens, collected immediately after the animal’s sacrifice, were transferred into refrigerated containers to the laboratory within 1 h. The porcine tissues were washed with an isotonic solution, and excess connective and adipose tissue was trimmed off. Finally, the samples were treated for 1 h with an isotonic solution containing trehalose 5% (*w*/*v*) and then stored at −80 °C for at least one week before use. For their use in ex vivo permeation studies, frozen tissue samples were washed with isotonic solution and subjected to thermal shock to separate the mucosal epithelium from the adipose and submucosal connective tissue. The latter was achieved by immersing samples for approximately 1 min in a preheated isotonic solution (60.0 ± 0.5 °C) and then manually separating the buccal mucosa from the underlying portion [29].

#### 2.15.2. Ex Vivo Permeation Assay

The obtained buccal mucosa was conditioned and washed in an isotonic solution overnight. Subsequently, it was equilibrated at room temperature and repeatedly washed with isotonic solution to remove any residual biological material that could interfere with further quantitative analyses. Appropriate portions of the mucosa were then cut and placed between the donor and the acceptor compartments of a Franz-type vertical diffusion cell (Permeagear, amber, unjacketed, flat flange joint, 11.28 mm orifice diameter, 8 mL acceptor volume, SES GmbH-Analysesysteme, Bechenheim, Germany) filled with ultrapure water and citrate buffer pH 5.5 containing β-cyclodextrin (3% *w*/*v*), respectively, [30]. Cells were conditioned for approximately 15 min at 37.0 ± 0.5 °C and then the solution in the donor compartment was removed and promptly replaced with the mLNP-RSV-PEG10K nanocomposite (either as a mini-tablet or as powder) soaked with 0.5 mL of ultrapure water. The system was maintained at 37.0 ± 0.5 °C and, for the evaluation of the RSV-containing nanocomposite, protected from light for a variable time, up to a maximum of 1 h. At predetermined time intervals, 0.4 mL aliquots were withdrawn from the acceptor fluid and immediately replaced with the same volume of fresh acceptor fluid to maintain the sink conditions. The collected samples were subjected to UV-Vis analysis. Each experiment was repeated three times, and the results are reported as means (n = 3) ± SE.

#### 2.15.3. Quantification of RSV and GA Entrapped into the Buccal Tissue

At the end of each permeation experiment, the Franz cells were disassembled and the buccal mucosa was washed, placed in a beaker containing 2 mL of methanol, and heated up to 60.0 ± 0.5 °C for 2 min. The extraction procedure was repeated twice. The extraction liquors were collected into a 5 mL volumetric amber flask and brought to volume with methanol. The amount of active extracted was determined using HPLC-DAD analysis, as reported below. Results are reported as means (n = 6) ± SE.

### 2.16. HPLC-DAD Method to Quantify RSV and GA

RSV and GA amounts were determined by HPLC-DAD analyses carried out using an Agilent 1260 Infinity Instrument (Agilent Technologies, Santa Clara, CA, USA) equipped with a Quaternary Pump G1311B, a Diode Array Detector 1260 Infinity II, an autosampler, a column oven, and a computer integrating apparatus (OpenLAB ChemStation 3D UV Workstation, Santa Clara, CA, USA). A measure of 20 μL of each sample was subjected to chromatographic separation using a reversed-phase column Ace^®^ Excel Super C18 (5 μm, 100 Å, size 125 × 4.60 mm; VWR International, Radnor, PA, USA) kept at 25 ± 1 °C as a stationary phase and a mobile phase consisting of a mixture of a water solution of TFA (0.1% *v*/*v*, indicated as solvent A) and acetonitrile (indicated as solvent B) according to the following gradient method: 0–2 min A:B = 90:10; 2–22 min from A:B = 90:10 to A:B = 5:95; 22–23 min A:B = 5:95; 23–25 min from A:B = 5:95 to A:B = 90:10; 25–27 min A:B = 90:10. The flow rate was set at 1 mL/min, the DAD considered range was 190–640 nm, and the evaluated chromatograms were those extracted at 250 nm (for GA quantification) and 305 nm (for RSV quantification). The following calibration curves were constructed:

- Six standard methanolic RSV solutions in the concentration range 0.25–10 μg/mL were injected. Retention time: 11.25 min; linear regression: area = 13,6745 x [mg/mL] (R = 0.999).
- Five standard methanolic GA solutions in the concentration range: 8–25 μg/mL were injected. Retention time: 21.26 min; linear regression: Area = 27,628.07 x + 3.54 [mg/mL] (R = 0.999).

Intraday and interday variations were lower than sensibility.

### 2.17. Data Analysis

The data are expressed as means ± standard error (SE). All differences were statistically evaluated with Student’s *t*-test or the one-way analysis of variance (ANOVA or F-test). Data were considered statistically significant when *p* < 0.05.

## 3. Results and Discussion

Preliminarily, the lipid mixture (MIX-RSV) to be used for the preparation of mLNP-RSV, which was analyzed in terms of RSV and GA content to evaluate their homogeneous distribution in the mixture and stability under the preparation conditions. As a result, the percentages of RSV and GA were 4.83 ± 0.08% (*w*/*w*) and 2.84 ± 0.08% (*w*/*w*), respectively, which are quite close to the theoretical ones (5.00% for RSV and 3.00% for GA), indicating that the use of high temperatures during the preparation of MIX-RSV did not result in significant degradation of either of the actives. Furthermore, as the results were greatly reproducible, the homogeneity of the preparation was confirmed. The lipid mixture was therefore suitable for the production of RSV-loaded mLNPs, which were prepared using the hot melt technique as a high-energy top-down method, according to the already optimized and published experimental conditions [19]. The resulting mLNPs were then evaluated in terms of the actives’ loading, particle size, PDI, and Z-potential (Table 1). The characteristics of the nanoparticles were in agreement with those reported in the previously published paper, confirming the reproducibility of the preparation method and procedure [19].

Considering all the issues related to a fluid formulation (e.g., stability, volume, transport, etc.), together with the limitations strictly connected to the post-extraction socket healing (e.g., administration), the mLNP-RSV were not directly useful as a dispersion, but they should be converted into a handle solid dosage form. A frequently used method for removing water from a colloidal dispersion is the freeze-drying technique, which would be highly advantageous given the well-known thermolability of RSV, and could lead to a dry, highly porous, hygroscopic, and easily re-dispersible powder. However, the freeze-drying of the dispersion in this manner results in the formation of ice crystals causing breakage, deformation, and aggregation of the lipid nanoparticles, making them difficult to redisperse or significantly altering the characteristics of the freshly prepared dispersion. To address this issue, cryoprotectants can be used. As already reported [19], standard cryoprotectants (sugars and polyols) were evaluated for freeze-drying the mLNP-RSV. However, these molecules are osmotically active, and this phenomenon is not always desirable. Indeed, both to protect the mLNP-RSV during the freeze-drying process and obtain a porous nanocomposite, seven different hydrophilic polymeric cryoprotectants were explored in different mLNP/polymer ratios as reported in Table S1 (see Supplementary Materials). However, some samples were unsuitable for the DLS measurements due to incomplete redispersion, leading to large solid particles floating (indicated as “*null*”).

HA was tested only at the minimum weight ratio selected, i.e., 1:0.5 *w*/*w*, as HA dispersions at higher concentrations were extremely viscous. However, it was not possible to analyze the samples containing HA, as the powder obtained was not redispersible, even by prolonged vortexing or sonicating. CMC was tested at three weight ratios: 1:0.5, 1:1, and 1:1.5 *w*/*w*. Similarly to HA, CMC dispersions at higher concentrations were excessively viscous and, therefore, unsuitable. Moreover, CMC was quickly discarded, as the dispersion of mLNP-RSV in the presence of CMC, at all tested weight ratios, appeared visibly coarse and was prone to rapid sedimentation. Furthermore, the redispersion process was slow or incomplete, even by vortexing or sonicating. As a result, parameters such as particle size, PDI, and Z-potential could not be evaluated. To evaluate PVA, PVP K30, PVP K90, PEG6K, and PEG10K, six weight ratios between the mLNP and each polymer were tested. PVA and PVP K90, at all the tested concentrations, caused the same issues already observed with CMC and were therefore discarded. The use of PVP K30 at the 1:5 and 1:8 weight ratios (*w*/*w*) allowed for a complete redispersion, although slow and achievable only by prolonged vortexing or sonicating. Moreover, as reported in Table 2, a significant increase in particle size and PDI compared to the native system is observable, likely related to the drastic reduction in Z-potential, which was close to zero. Consequently, PVP K30 was also discarded. Both PEG6K and PEG10K, when tested at the three lowest weight ratios, led to unsatisfactory results. However, the three highest weight ratios resulted in the complete redispersion of the mLNP-RSV, yielding colloidal dispersions having the characteristics shown in Table 2. The use of PEG6K still caused an increase in particle size and PDI, although the Z-potential values remained negative and not significantly different from the native dispersions. PEG10K appeared to have the best cryoprotective effect, resulting in a minimal increase in terms of mLNP-RSV particle size after redispersion following freeze-drying. Thanks to its higher molecular weight, it likely better separates the nanoparticles, even during the concentration phase of the sublimation process, minimizing interaction and growth particles phenomena. The ability of both PEGs to effectively interact with mLNP-RSV and be effective as cryoprotectants may be correlated to their affinity with the components of the nanoparticles themselves. Indeed, LB, the liquid lipid used to prepare the MIX-RSV, is a mixture that contains mono- and di-esters of fatty acids with PEG-8 and free PEG-8. It is therefore likely that the PEG chains of the LB, in the three-dimensional rearrangement of lipids during the formation of mLNPs, are predominantly exposed on the external surface, where they can interact with the PEG chains of cryoprotectants.

**Figure 1:**
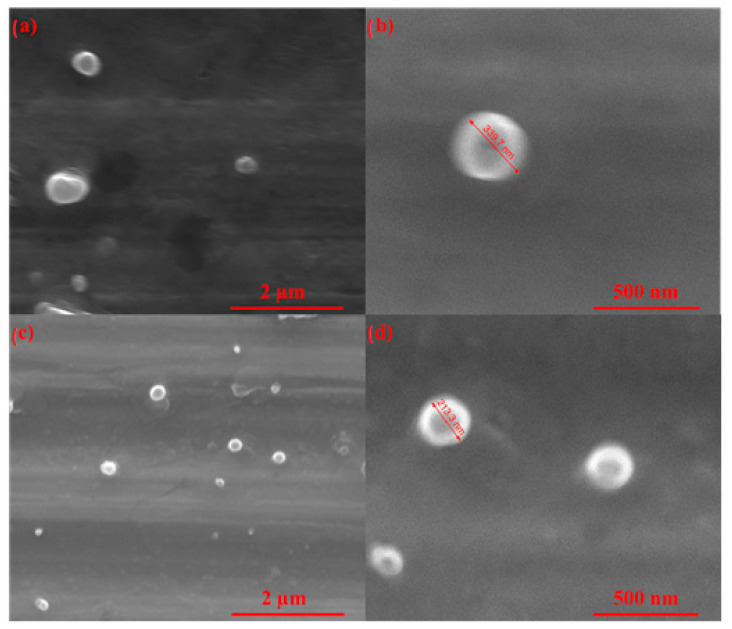
SEM micrographs of mLNP-RSV-P6K at (**a**) 40,000× and (**b**) 150,000× magnification and of mLNP-RSV-P10K at (**c**) 40,000× and (**d**) 150,000× magnification.

Considering the particle size of the redispersed nanoparticles as well as the ease and speed of the redispersion process, the best selected samples were mLNP-P6K (F) and mLNP-P10K (F). These samples were then further subjected to SEM analysis after reconstitution in water and appropriate dilution in order to confirm the DLS results. In the following analyses the two selected nanocomposites were indicated as mLNP-RSV-P6K and mLNP-RSV-P10K for the reader’s convenience. In Figure 1, the morphology of the redispersed mLNPs is displayed.

According to SEM pictures, spherical, homogeneous, and nanometric particles were observed, independent of the molecular weight of the PEG used as cryoprotectant. Notably, no crystals were observed, suggesting the stability of the composite mixture ever after the redispersion in water. In addition, it is important to mention that the diameter of the mLNP-RSV-P10K (d ~ 210 nm) is smaller than that obtained for mLNP-RSV-P6K (d ~ 340 nm), in agreement with DLS findings.

Consequently, both PEG-based nanocomposites were further characterized by XRD, FTIR, Raman spectroscopy, and thermal analysis.

**Figure 2:**
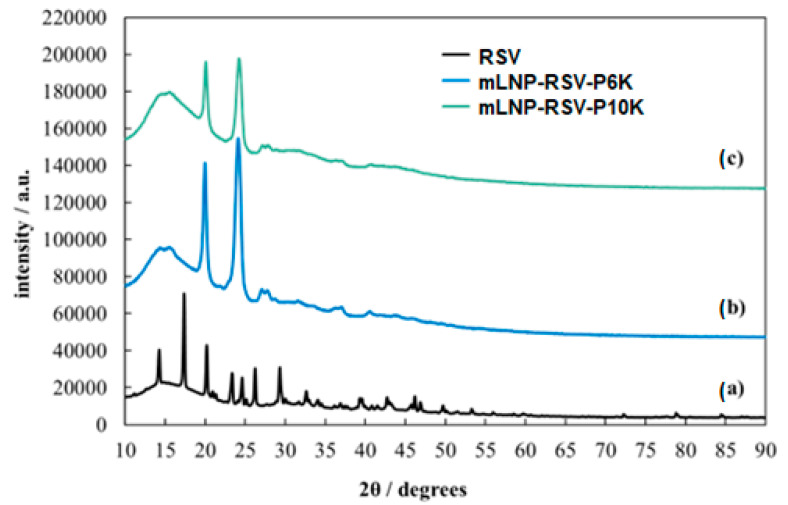
XRD pattern of (**a**) RSV, (**b**) mLNP-RSV-P6K, and (**c**) mLNP-RSV-P10K.

The physical state of raw RSV, mLNP-RSV-P6K, and mLNP-RSV-P10K nanocomposites was evaluated by X-ray diffraction microscopy, as shown in Figure 2.

The XRD pattern of RSV displays several intense diffractions peaks, ranging from 15 to 30 °C (2θ angles 14.34°, 17.38°, 20.22°, 23.42°, 24.68°, 26.16° and 29.36°) and a few minor peaks in the range 30–50° degrees, supporting its crystalline structure [31]. Notably, the reflection peaks of the mLNPs do not fit the XRD pattern of raw RSV. Indeed, the two main reflection peaks at 2θ angles 20.01 and 23.95° and a few minor peaks at 2θ equals to 27.16°, 27.78°, 37.29°, and 46.65° are consistent with the presence of PEGs [32,33]. This finding suggests the loss of RSV crystallinity after its embedding into the lipid mixture and in the nanoparticles, also supported by a broad and diffuse peak at 15°, consistent with a heterogeneous nanodispersed amorphous structure.

**Figure 3:**
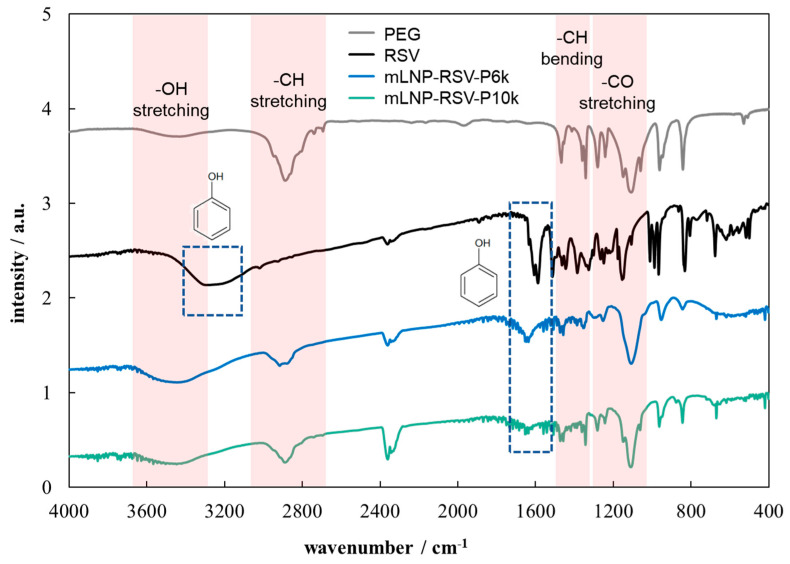
FTIR spectra of PEG, RSV, mLNP-RSV-P6K, and mLNP-RSV-P10K.

FT-IR and Raman spectra were then recorded in order to investigate, whereas the used trans-RSV still remained in this biologically active isomeric form. In Figure 3, the FT-IR spectra of RSV, mLNP-RSV-P6K, and mLNP-RSV-P10K are displayed.

According to Figure 3, a broad peak at around 3260 cm^−1^ is displayed for RSV, in agreement with the presence of phenolic hydroxyl groups in the latter (i.e., O-H stretching vibration). Moreover, peaks ranging from 1600 to 1444 cm^−1^ are due to the skeletal vibration of the benzene ring, those at 1384 and 1147 cm^−1^ are related to C-O stretching vibration, while the absorption peaks at 965 and 986 cm^−1^ are attributed to the bending of the C=C bond, as expected for trans-RSV [25,31,32,34].

In the case of the nanocomposites, some of the relevant peaks of RSV are visible. Indeed, absorption peaks at 1510, 1461, 1145, and 965 cm^−1^ in both the nanocomposites are visible. It is noteworthy that the peak at 965 cm^−1^, typical of the trans- -C=C- bending vibration characteristic of the trans-RSV, is also visible in the FT-IR spectra of the nanocomposites, independent of the polymer molecular weight. This finding supports the stability of RSV during all the phases leading to nanocomposites preparation [31]. Conversely, the peaks at ~3440 cm^−1^ (namely, O-H stretching vibration), ~2890 cm^−1^ (i.e., C-H vibration mode), 1460, and 1340 cm^−1^ (i.e., C-H bending vibrations) and those between 1280 and 1100 cm^−1^ (i.e., C-O-H stretching vibrations) can be attributed to the presence of PEGs in the mixture [33]. Analogous results were obtained for the two nanocomposites. Notably, the peak at ~2360 cm^−1^ is assigned to the asymmetric stretching of CO_2_.

**Figure 4:**
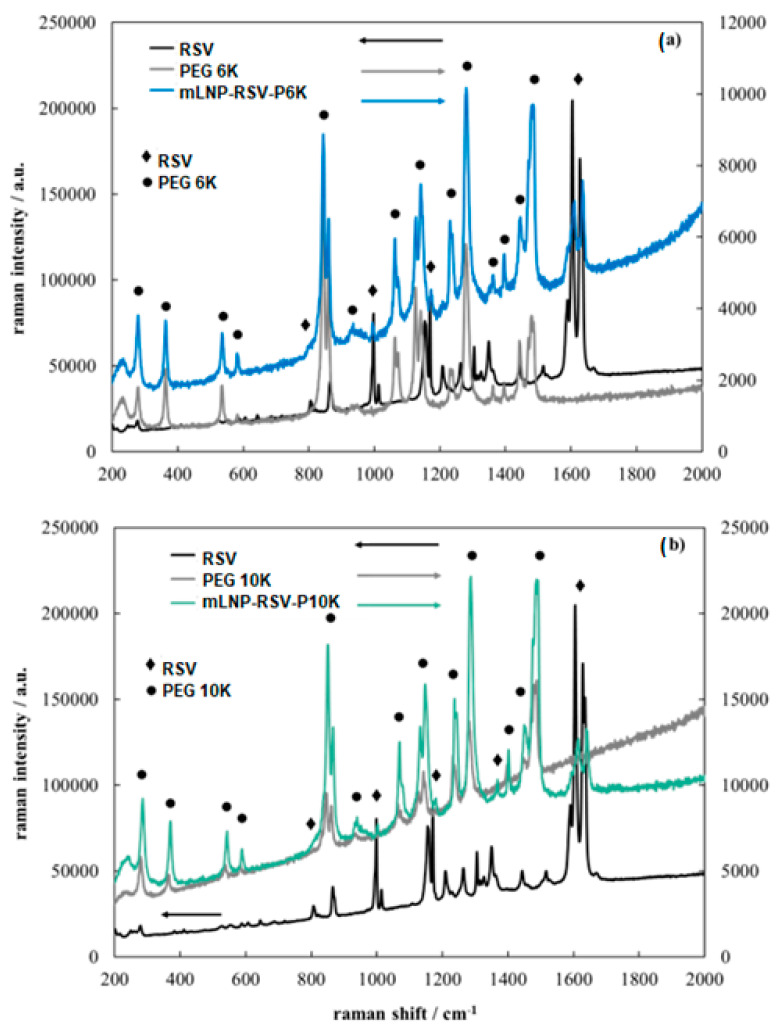
Raman spectra of (**a**) mLNP-RSV-P6K and (**b**) mLNP-RSV-P10K.

In Figure 4, the Raman spectra are reported. The Raman spectra were also carried out for pure PEG and pure RSV in order to have a reference.

The strong bands at 1628 and 1602 cm^−1^ in the nanocomposites’ spectra can be related to vibration of C=C and C-H bonds, respectively, as expected for the trans-olefin carbon, while the peak at 1596 cm^−1^ is assigned to the vibration of the phenyl rings. Moreover, the peaks at 809 cm^−1^ and 996 cm^−1^ can be related to vibrations from the p-OH and m-OH ring, respectively, while that at 1174 cm^−1^ can be assigned to C-H vibrations of OH ring [35]. The presence of trans-RSV in the nanocomposite is also supported by the lack of peak splitting in both Raman and FT-IR spectra as observed in Gupta et al. [25] for trans isomerization to cis-RSV.

**Figure 5:**
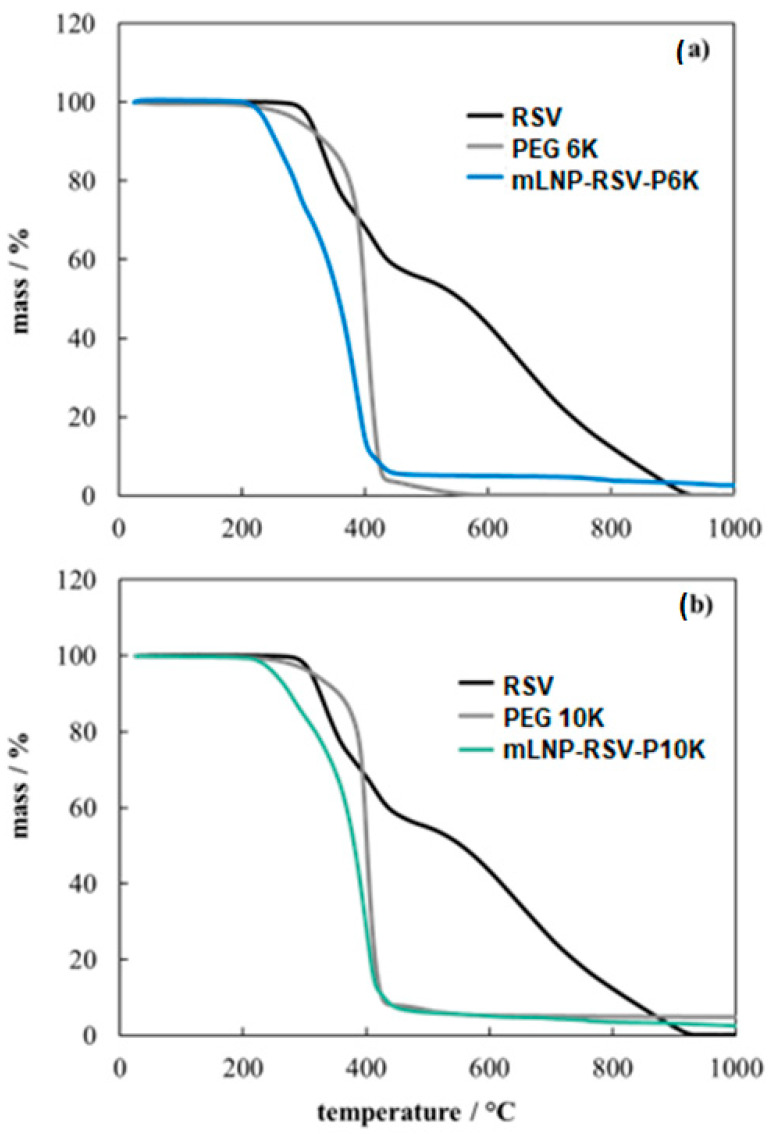
TG curves in dynamic dry air for (**a**) mLNP-RSV-P6K and (**b**) mLNP-RSV-P10K compared with RSV and PEG6K pure compounds.

In Figure 5, TG curves related to RSV as a pure compound and nanocomposites are reported. Notably, TG curves for pure PEG were also reported in order to better understand the behavior of the mixtures.

For all the investigated samples, the initial weight loss up to 200 °C can be related to water elimination. The TG curve of RSV reveals a sharp endothermic peak at 270 °C, associated with the onset of melting of the sample item [31]. Indeed, no mass loss was evidenced at this temperature (see Supplementary Materials, Figure S1). Moreover, RSV appears stable until 240 °C, after which two consecutive decomposition steps were found. In particular, a first mass loss of about 44.64% occurs in the range of 200–500 °C while the material is totally decomposed during the second decomposition step, where a mass loss of 55.30% was estimated in the range 500–1000 °C. Both phenomena are coupled to an exothermic behavior with a single small peak at around 550 °C (see Supplementary Materials, Figure S1). These findings were also supported by the literature [36].

In the case of pure PEG6K, an endothermic peak at around 68 °C is present, corresponding to the melting point of the polymer [32]. The same was found for PEG10K (see Supplementary Materials, Figure S2). Notably, TG curves for both polymers contain only one mass loss step, according to Figure 5.

Considering the nanocomposites, TG curves reveal that their decomposition occurs at a lower temperature than that of pure RSV. Nevertheless, the nanocomposites display very good thermal stability up to ~230 °C. It is important to mention that the endothermic peak of RSV cannot be observed when it is embedded into the whole nanocomposite (see Supplementary Materials, Figure S3). Indeed, the endothermic peak in the DTA curve at about 70 °C for the PEG6K-based nanocomposite corresponds to the melting point of pure PEG6K. Moreover, no marked thermal events were observed during the mixture heating, suggesting the formation of an amorphous system. The same findings were obtained for mLNP-RSV-P10K. For the latter, it is noteworthy to mention that the mixture started to decompose a little bit after the mLNP-RSV-P6K nanocomposite, suggesting a slightly higher thermal stability.

**Figure 6:**
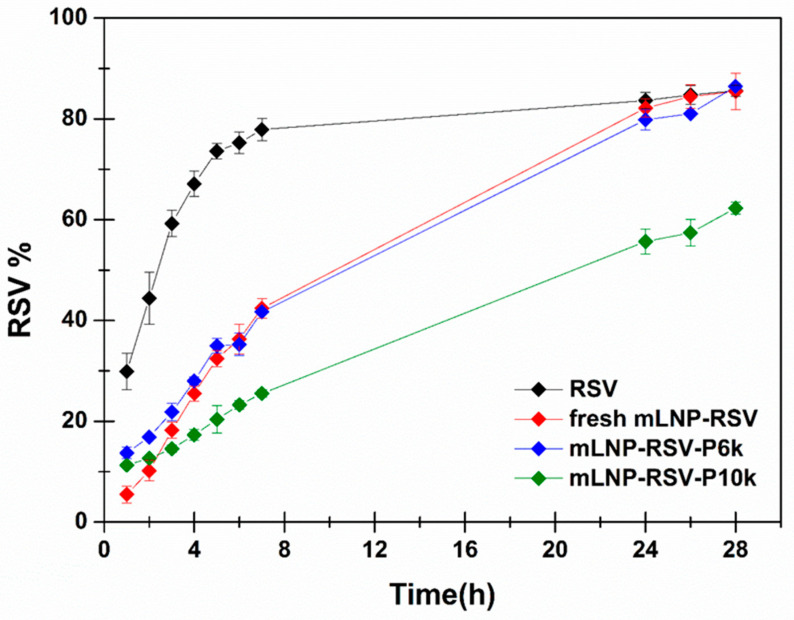
Percentage of RSV partitioned into the lipophilic acceptor compartment as a function of incubation time when loading into the donor compartment: free RSV as a solution (black), fresh mLNP-RSV dispersion (red), redispersed mLNP-RSV-P6K nanocomposite (blue), and redispersed mLNP-RSV-P10K nanocomposite (green).

For the purpose of promoting alveolar wound healing, the solid nanocomposites should be applied into the post-extraction cavity. Here, the presence of an extremely hydrophilic PEG matrix will ensure the rapid and complete dispersion of these powders once they come into contact with the physiological fluids such as wound exudates, oral cavity secretions, etc., while the therapeutic activity of the mLNP-RSV will be exerted following their partitioning from the site of administration (comparable to a hydrophilic compartment) to the surrounding tissues (comparable to a lipophilic compartment). Once they reach the target tissues, the mLNP-RSV can interact with the local cells, enabling in situ delivery the loaded RSV and promoting the complex process of tissue regeneration while simultaneously supporting the asepsis of the wound. To assess the ability of the nanocomposites to release the mLNP-RSV otherwise trapped within the hydrophilic polymer, in vitro partition studies were conducted from a hydrophilic compartment to a lipophilic one. To this aim, the modified Transwell method was used [27]. Transwell inserts with a cut-off of 0.45 μm were selected as they allow the passage of the mLNP-RSV as a whole. 1-octanol was then chosen as the acceptor fluid as it is a widely used hydrophobic liquid able to mimic the characteristics of the naturally occurring cell membrane lipids. As reported in Figure 6, free RSV rapidly reaches the lipophilic compartment, to which it is greatly affine. Indeed, after 8 h, 80% of the starting RSV dose loaded into the donor chamber was already detected in the 1-octanol compartment, reaching a plateau. The results obtained with the mLNP-RSV, however, are significantly different. In particular, it can be observed that, at early time points, the partitioning of fresh mLNP-RSV appears much slower than that of mLNP-RSV redispersed after freeze-drying, considering both the studied nanocomposites. This can be attributed to the even slight increase in particle sizes previously observed. As the mLNP-RSV obtained by redispersion of the PEGs-based nanocomposite are larger than the fresh one, they may be more affected by sedimentation phenomena, bringing them into contact with the exchange surface between the two compartments more quickly, thereby accelerating the partitioning process. Overall, by analyzing the redispersed mLNP-RSV-P10K nanocomposite, the slowest partitioning rate was observed. This is likely due to the presence of long PEG10K chains, which entrap the lipid nanoparticles and modulate their migration toward the lipophilic compartment. In contrast, aside from the initial time points, the freshly prepared mLNP-RSV dispersion and the reconstituted mLNP-RSV-P6K nanocomposite yielded comparable results. It is evident, therefore, that the use of a lower MW PEG is less able to create a hydrophilic network toward the nanoparticles, resulting in reduced effectiveness as cryoprotectant (the particle size after redispersion is higher than that obtained by using PEG10K), but also in the creation of a weaker hydrophilic external network that opposes nanoparticles’ movement. Furthermore, it is likely that any delay in the migration of mLNP-RSV nanoparticles from the redispersed PEG6K-based nanocomposite due to the presence of the PEG6K external matrix is compensated by the increased particle size, which promotes sedimentation through the polymeric matrix itself. The results obtained highlight that both nanocomposites could be effective as innovative delivery systems for RSV. In particular, the slowing effect on mLNP-RSV partitioning observed when studying the PEG10K-based nanocomposite could represent an additional advantage, allowing modulation of the therapeutic effect and ensuring a prolonged therapeutic coverage period.

The obtained results led to the selection of the PEG10K-based nanocomposite as a starting point to further obtain a hybrid nanocomposite enriched in hydroxyapatite (HXA). Several PEG10K:HXA weight ratios were tested and also two different methods of HXA incorporation were evaluated:

1. Mixing the freeze dried mLNP-RSV-P10K with HXA powder (indicated as mLNP-RSV-P10K-HXA-1);
2. Preparing a HXA suspension that is added into the PEG10K dispersion to be mixed with the mLNP-RSV prior to the freeze-drying process (indicated as mLNP-RSV-P10K-HXA-2).

In both cases, the obtained white powders were firstly subjected to a visual redispersion assessment: by gently mixing, the suspensions were immediately reconstituted, and appeared as before the freeze-drying process. Furthermore, the powders were also manipulated to prepare the hybrid nanocomposite mini-tablets as an easy to handle, standardized (in terms of weight, volume and amount of actives), and reproducible dosage form to be directly inserted into the post-extraction cavity instead of the voluminous and spongy powders obtained by freeze-drying. The mini-tablet preparation enabled the selection of the best PEG:HXA ratio as the higher ratios (1:1.25 and 1:1.5 *w*/*w*) were discarded due to hardness and resistance to compression of powder obtained. Thus, the best selected ratio was the PEG:HXA = 1:1 *w*/*w* one. The latter led to the incorporation of the maximum amount of HXA while keeping the material soft and easy to be compressed.

Both the hybrid powder and the mini-tablets obtained using the selected PEG:HXA ratio were then characterized and compared to the starting HXA-free nanocomposite.

The two powder nanocomposites were first evaluated in terms of drug loading % in comparison to the HXA-free nanocomposite. As reported in Table 3, the DL% values of both hybrid nanocomposites were equal to each other and lower than the HXA-free nanocomposite (not-hybrid nanocomposite). This is clearly due to the contribution of HXA, which increases the mass of the nanocomposite, thus reducing the nanoparticles’ content per weight unit. These results indicated that the method of HXA insertion did not affect the actives’ loading nor the amount of HXA incorporated. Furthermore, the nanocomposites homogeneity was proven by the extremely low variability of the results.

The flow properties of the hybrid and not-hybrid nanocomposite powders were then evaluated both indirectly and directly. The indirect method, according to *FU XII ed.*, consisting of the evaluation of the nanocomposites’ density, is useful to calculate the Hausner ratio (H) and the Carr index %. The first highlighted differences between the nanocomposites related to their density (Table 4). The mLNP-RSV-P10K-HXA-1 nanocomposite was characterized by both bulk and tapped densities which were 2 to 3-folds higher than the other nanocomposites. This could be attributed to the preparation procedure, consisting of mixing two different powders instead of directly freeze-drying the whole mass. Apart from this, no great differences were highlighted in terms of theoretical flow properties, as the H and Carr index % values were almost similar for the three powders which were all predicted to possess poor or barely acceptable flow characteristics. To experimentally determine the flow properties, the angle of repose was evaluated in accordance with *FU XII ed.* As reported (Table 4), the presence of the HXA modified the flow properties of the mLNP-RSV-P10K nanocomposite, worsening them. Particularly, the addition of HXA before the freeze-drying process (mLNP-RSV-P10K-HXA-2) led to a non-flowing powder (Φ > 40°).

**Figure 7:**
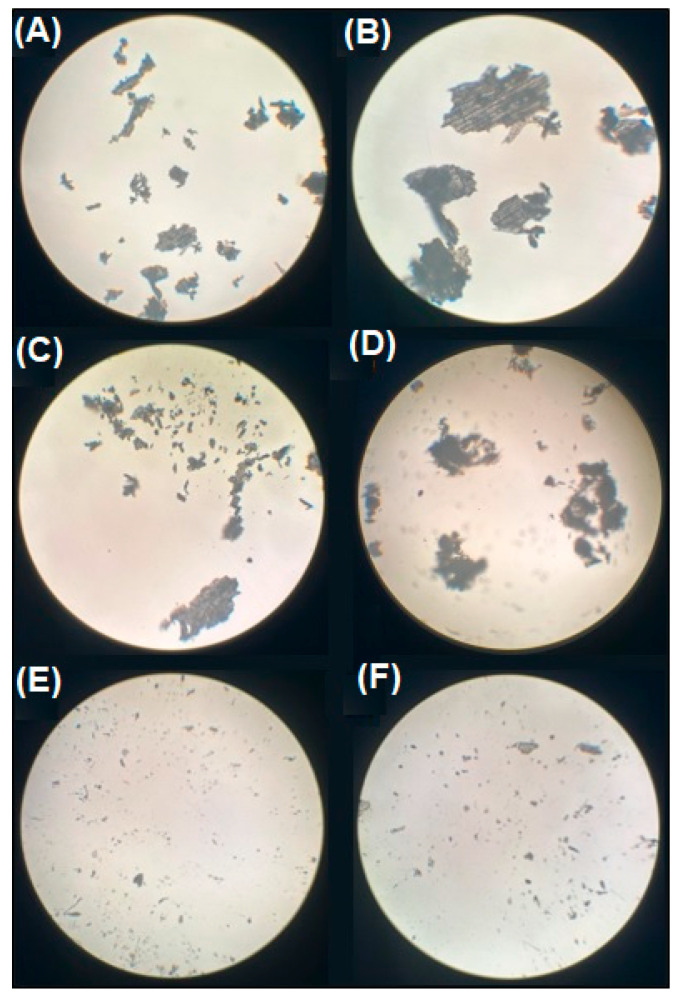
Photographs of mLNP-RSV-P10K powder at (**A**) 4× and (**B**) 10× magnification; mLNP-RSV-P10K-HXA-1 powder at (**C**) 4× and (**D**) 10× magnification; and mLNP-RSV-P10K-HXA-2 powder at (**E**) 4× and (**F**) 10× magnification.

The apparent discrepancy observed between the theoretical evaluation and the experimental results are likely related to the geometry of the powder particles, as confirmed by the optical microscopy investigations (Figure 7). Additionally, it is evident that the HXA-containing nanocomposites are characterized by smaller powder particles, particularly the mLNP-RSV-P10K-HXA-2 one. This observation is consistent with the data obtained from the angle of repose assay.

These hybrid and not-hybrid nanocomposite powders were then used to prepare the mini-tablets, possessing the characteristics highlighted in Table 5. As reported, the preparation method gave consistent results in terms of reproducibility.

To choose the most suitable nanocomposite form (powder vs. mini-tablet) to be inserted into the post-extraction socket, further experiments were carried out. Firstly, the disintegration assay, once in contact with an aqueous medium, was carried out. To better simulate the in vivo conditions, a mini-tablet or 30 mg of (free) powder was inserted into a glass crucible and soaked with a small volume of aqueous solution prewarmed at 37.0 ± 0.5 °C. The time required to observe the complete disintegration of the mini-tablet/redispersion of the powder was recorded and indicated as DT (Table 6).

As observable, the HXA presence did not significantly affect the redispersion behavior of the free powder, while it drastically decreased the DT of the mini-tablets, reducing it 5–7 fold. As expected, the free powder form redispersed faster than the mini-tablet disintegration occurred as the contact surface with the aqueous medium is far larger when the nanocomposites are in powder form.

**Figure 8:**
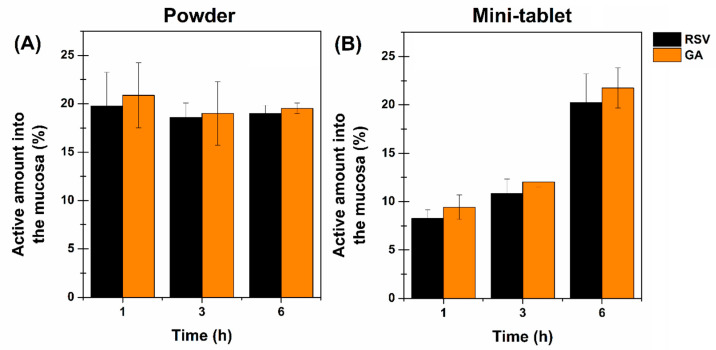
Percentage dose of RSV (black) and GA (orange) accumulated into the buccal mucosa loaded into the donor compartment as a function of incubation time when evaluating (**A**) the mLNP-RSV-P10K free powder and (**B**) the mLNP-RSV-P10K mini-tablet. Means (n = 6) ± SE.

Finally, ex vivo evaluations were performed in order to verify the ability of the not-hybrid nanocomposite, both as a powder and as a mini-tablet, to ensure the high interaction of actives with and accumulation into the target tissue. The choice to perform the ex vivo experiments only for the mLNP-RSV-P10K nanocomposite (either as powder or mini-tablet) was caused by preliminary experimental set-up experiences that highlighted the HXA precipitation on the tissue surface, which would have led to misleading results. As a model of membrane able to mimic the in vivo tissue, the buccal porcine mucosa was selected. Results are graphically presented in Figure 8 as the percentage amount of RSV and GA accumulated as a function of time respect to the total amount of each active initially loaded into the donor compartment of the Franz diffusion cell. It is worth pointing out that neither RSV nor GA were detected in the acceptor chamber, even after 6 h of the experiment. Nevertheless, as desirable, both actives tended to accumulate into the buccal tissue over time. As observable, the maximum percentage of both actives entrapped into the mucosa was around 20% of the initially administered dose, both when evaluating the free powder (Figure 8A) and the mini-tablet (Figure 8B). This might suggest that this is the maximum amount which could be entrapped into the considered surface (1 cm^2^) in the employed experimental set-up. This hypothesis is further supported by the results showing the same amount of both RSV and GA accumulated into the mucosa after 1, 3, and 6 h of incubation when the free powder was administered. On the other hand, the different behavior observed for the mini-tablet, capable of inducing a time-dependent increase in the entrapment of the actives in the mucosa, could be correlated to the significant differences in the DT values of the formulations previously observed; in fact, the mini-tablet required longer contact time with the aqueous medium to disintegrate, thus releasing the mLNP-RSV which could interact with the target tissue, allowing the actives to accumulate. In contrast, the powder is quickly dispersed once soaked.

## 4. Conclusions

In this work, several steps have been carried out to finally propose a novel hybrid nanocomposite delivery system potentially able to promote alveolar healing following tooth extraction by matching the potentiality of RSV and HXA. To stabilize and locally deliver RSV, it was successfully embedded into multicomponent lipid nanoparticles containing also glycyrrhetinic acid and menthol as functional hydrophobic components able to support the antioxidant properties of RSV and promote its interaction with the target tissue. To merge the benefit of a local treatment with both RSV (anti-inflammatory and antioxidant activities useful for wound healing purposes) and HXA (bone substitute often used to avoid alveolar ridge reduction), PEG10K was used as the hydrophilic and biocompatible polymer matrix. Indeed, it resulted in being able to act as a cryoprotectant for the mLNP-RSV nanoparticles while also homogeneously suspending the HXA, thus resulting in a soft and handy hybrid matrix powder following freeze-drying. Considering the various PEG:HXA ratios evaluated, and the HXA-incorporation methods experienced, the mLNP-RSV-P10K-HXA-1 hybrid nanocomposite emerged as the best one. Then, the hybrid nanocomposite in powder form was compared to the mini-tablet one to assess the best solution to address the main purpose of the work. The results clearly showed that CPR-HXA-1 was the best choice as an innovative delivery system that was directly loadable into the post-extraction socket as it is characterized by a 58.9 mm^3^ volume, which is compliant with the dimensions of the post-extraction cavity [37]. In contrast, the same amount of the free powder occupies a volume of 187.5 mm^3^, only allowing the use of a low dose of nanocomposite. Furthermore, the mini-tablet allows the dose of the administered drug to be standardized and gives the operator time to suture the wound thanks to the time needed for tablet disintegration.

To conclude, this study effectively served to develop a promising nanocomposite and to identify the theoretical best dosage form in which it should be administered to address the highlighted problem of alveolar healing following tooth extraction. Investigating it’s in vivo osteoinductive and wound healing effects will then represent the next phase of this research.

## Figures and Tables

**Table 1:** Characteristics of the mLNP-RSV. Means (n = 9) ± SE.

**DR%**	*RSV*	98.35 ± 0.88
	*GA*	98.41 ± 0.73
**DL%**	*RSV*	4.69 ± 0.01
	*GA*	2.64 ± 0.01
**LE%**	*RSV*	95.68 ± 0.07
	*GA*	97.16 ± 0.20
**Diameter (nm)**	165.23 ± 2.96	
**PDI**	0.259 ± 0.012	
**Z-potential (mV)**	−20.88 ± 6.59	

**Table 2:** Characteristics in terms of particle size (nm), PDI, Z-potential and redispersibility of freeze-dried nanocomposites prepared using different weight ratios between the mLNP and polymers. Means (n = 3) ± SE.

Formula Code	Polymer	mLNP/Polymer Ratio (*w*/*w*)	Diameter (nm)	PDI	Z-Potential (mV)	Observations
mLNP-K30 (E)	PVP K30	1:5	458.95 ± 165.65	0.697 ± 0.248	−1.03 ± 0.87	Slow redispersion. Vortex/ultrasound needed
mLNP-K30 (F)		1:8	635.95 ± 203.95	0.852 ± 0.148	−1.56 ± 0.87	Slow redispersion. Vortex/ultrasound needed
mLNP-P6K (D)	PEG6K	1:3	409.65 ± 70.75	0.782 ± 0.011	−26.25 ± 0.75	Slow redispersion. Vortex/ultrasound needed
mLNP-P6K (E)		1:5	392.76 ± 85.68	0.517 ± 0.060	−18.89 ± 3.77	Slow redispersion. Vortex/ultrasound needed
mLNP-P6K (F)		1:8	475.43 ± 69.67	0.573 ± 0.042	−19.76 ± 3.53	Fast and easy redispersion
mLNP-P10K (D)	PEG10K	1:3	376.73 ± 91.22	0.518 ± 0.111	−19.33 ± 4.40	Slow redispersion. Vortex/ultrasound needed
mLNP-P10K (E)		1:5	255.22 ± 31.47	0.618 ± 0.096	−18.77 ± 3.06	Slow redispersion. Vortex/ultrasound needed
mLNP-P10K (F)		1:8	285.69 ± 28.13	0.582 ± 0.067	−18.93 ± 3.05	Fast and easy redispersion

**Table 3:** Characteristics of the hybrid and not-hybrid nanocomposite powders in terms of DL% of both RSV and GA. Means (n = 9) ± SE.

Sample	DL%	
	RSV	GA
**mLNP-RSV-P10K**	0.44 ± 0.02	0.24 ± 0.01
**mLNP-RSV-P10K-HXA-1**	0.28 ± 0.01	0.15 ± 0.01
**mLNP-RSV-P10K-HXA-2**	0.28 ± 0.01	0.15 ± 0.01

**Table 4:** Bulk properties of the hybrid and not-hybrid nanocomposite powders in terms of density (n = 3), Hausner ratio (H), Carr index (%), and angle of repose (Φ, n = 6). Means ± SE.

Sample	Density (g/mL)		H	Carr Index (%)	Φ (°)
	ρ_B_	ρ_T_			
mLNP-RSV-P10K	0.053 ± 0.003	0.074 ± 0.001	1.4	28.4%	26.09 ± 0.45°
mLNP-RSV-P10K-HXA-1	0.160 ± 0.032	0.210 ± 0.015	1.3	24.0%	31.83 ± 0.40°
mLNP-RSV-P10K-HXA-2	0.077 ± 0.005	0.099 ± 0.001	1.3	22.7%	45.62 ± 0.17°

**Table 5:** Characteristics of the hybrid and not-hybrid nanocomposite mini-tablets: weight, diameter, thickness, and content of RSV and GA per tablet. Means (n = 6) ± SE.

Formula Code	Starting Nanocomposite	Weight (mg)	Diameter (mm)	Thickness (mm)	RSV (μg)	GA (μg)
CPR	mLNP-RSV-P10K	29.83 ± 0.13	5	3	131.2 ± 0.6	71.6 ± 0.3
CPR-HXA-1	mLNP-RSV-P10K-HXA-1	29.85 ± 0.13	5	3	83.6 ± 0.4	44.8 ± 0.2
CPR-HXA-2	mLNP-RSV-P10K-HXA-2	30.10 ± 0.27	5	3	84.3 ± 0.8	45.2 ± 0.4

**Table 6:** Disintegration time of the hybrid and not-hybrid nanocomposites as free powders or mini-tablets. Means (n = 3) ± SE.

Nanocomposite	DT (min)	
	Free Powder	Mini-Tablet
**mLNP-RSV-P10K**	1.36 ± 0.04	22.37 ± 0.62
**mLNP-RSV-P10K-HXA-1**	1.16 ± 0.04	3.24 ± 0.10
**mLNP-RSV-P10K-HXA-2**	0.73 ± 0.15	4.14 ± 0.05

## Data Availability

The original contributions presented in this study are included in the article/Supplementary Materials. Further inquiries can be directed to the corresponding author.

## Supplementary Materials

The following supporting information can be downloaded at https://www.mdpi.com/article/10.3390/pharmaceutics17010112/s1, Table S1: Characteristics in terms of particle size (nm), PDI, Z-potential and redispersibility of nanocomposites prepared using different weight ratios between the mLNP and seven polymers. Means (n = 3) ± SE; Figure S1: Simultaneous TG/DTA curves for pure RSV; Figure S2: DTA curves for PEG6K and PEG10K; Figure S3: Simultaneous TG/DTA curves for (a) mLNP-RSV-P6K and (b) mLNP-RSV-P10K.

## Abbreviations

CD—cyclodextrin; CMC—carboxymethyl cellulose; DL%—drug loading %; DLS—dynamic light scattering; DPPH—1,1-diphenyl-2-picrylhydrazil; DR%—drug recovery %; FTIR—Fourier transform infrared; FU XII ed—Italian official pharmacopeia XII ed.; GA—glycyrrhetinic acid; GMS—glyceryl monostearate 55–60; H—Hausner ratio; HA—hyaluronic acid sodium salt; HPLC-DAD—high-performance liquid chromatography-diode array detector; HXA—hydroxyapatite; LB—Labrasol^®^; LE%—loading efficacy %; ME—menthol; MIX-RSV—lipid mixture loaded with resveratrol; mLNP—multicomponent lipid nanoparticles; mLNP-RSV—multicomponent lipid nanoparticles loaded with resveratrol; MW—molecular weight; NLC—nanostructured lipid carriers; PDI—polydispersity index; PEG—polyethylene glycol; PVA—polyvinyl alcohol; PVP—polyvinylpyrrolidone; ROS—reactive oxygen species; RSV—resveratrol; SEM—scanning electron microscopy; SLN—solid lipid nanoparticles; TFA—trifluoroacetic acid; UV-Vis—ultraviolet-visible; V_B_—bulk volume; V_T_—tapped volume; XRD—X-ray diffraction.
