# Supplementary material for: Hybrid Nanocomposite Mini-Tablet to Be Applied into the Post-Extraction Socket: Matching the Potentialities of Resveratrol-Loaded Lipid Nanoparticles and Hydroxyapatite to Promote Alveolar Wound Healing
Source: Pharmaceutics. 2025 Jan 15;17(1):112. doi: 10.3390/pharmaceutics17010112 (PMC11769172; doi:10.3390/pharmaceutics17010112)
Supplement: Supplementary file 1 [file pharmaceutics-17-00112-s001.zip › pharmaceutics-3396427-supplementary.pdf]

pharmaceutics

Supplementary Materials

Hybrid Nanocomposite Mini-Tablet to Be Applied into the Post-Extraction Socket: Matching the Potentialities of Resveratrol-Loaded Lipid Nanoparticles and Hydroxyapatite to Promote Alveolar Wound Healing

Viviana De Caro [1], Giada Tranchida [2], Cecilia La Mantia [1], Bartolomeo Megna [2], Giuseppe Angellotti [3] and Giulia Di Prima [1,*]

[1] Dipartimento di Scienze e Tecnologie Biologiche Chimiche e Farmaceutiche (STEBICEF), University of Palermo, Via Archirafi 32, 90123, Palermo, Italy; viviana.decaro@unipa.it (V.D.C.); cecilialamantia000@gmail.com (C.L.M.)
[2] Dipartimento di Ingegneria, University of Palermo, Viale delle Scienze, 90128 Palermo, Italy; giada.tranchida@unipa.it (G.T.); bartolomeo.megna@unipa.it (B.M.)
[3] Istituto per lo Studio dei Materiali Nanostrutturati, Consiglio Nazionale delle Ricerche (ISMN-CNR), Via Ugo La Malfa 153, 90146 Palermo, Italy; giuseppe.angellotti@cnr.it
* Correspondence: giulia.diprima@unipa.it; Tel.: +39–09123896826

Data reported as supplementary materials:

- Table S1. Characteristics in terms of particle size (nm), PDI, Z-potential and redispersibility of nanocomposites prepared using different weight ratios between the mLNP and seven polymers. Means (n=3) ± SE.
- Figure S1. Simultaneous TG/DTA curves for pure RSV;
- Figure S2. DTA curves for PEG6K and PEG10K;
- Figure S3. Simultaneous TG/DTA curves for a) mLNP-RSV-P6K and b) mLNP-RSV-P10K.

Table S1. Characteristics in terms of particle size (nm), PDI, Z-potential and redispersibility of nanocomposites prepared using different weight ratios between the mLNP and seven polymers. Means (n=3) ± SE. Samples unsuitable for DLS measurements due to incomplete redispersion leading to large solid particles floating were indicated as "*null*".

Formula Code	Polymer	mLNP:polymer ratio (w/w)	Diameter (nm)	PDI	Z-potential (mV)	Observations
mLNP-HA	HA	1:0.5	*null*	*null*	*null*	Not redispersable
mLNP-CMC (A)	CMC	1:0.5	*null*	*null*	*null*	Not redispersable
mLNP-CMC (B)		1:1	*null*	*null*	*null*	Not redispersable
mLNP-CMC (C)		1:1.5	*null*	*null*	*null*	Not redispersable
mLNP-PVA (A)	PVA	1:0.5	*null*	*null*	*null*	Not redispersable
mLNP- PVA (B)		1:1	*null*	*null*	*null*	Not redispersable
mLNP- PVA (C)		1:1.5	*null*	*null*	*null*	Not redispersable
mLNP- PVA (D)		1:3	*null*	*null*	*null*	Not redispersable
mLNP- PVA (E)		1:5	*null*	*null*	*null*	Not redispersable
mLNP- PVA (F)		1:8	*null*	*null*	*null*	Not redispersable
mLNP-K30 (A)	PVP K30	1:0.5	*null*	*null*	*null*	Not redispersable
mLNP-K30 (B)		1:1	*null*	*null*	*null*	Not redispersable
mLNP-K30 (C)		1:1.5	*null*	*null*	*null*	Not redispersable
mLNP-K30 (D)		1:3	*null*	*null*	*null*	Not redispersable
mLNP-K30 (E)		1:5	458.95 ± 165.65	0.697 ± 0.248	-1.03 ± 0.87	Slow redispersion. Vortex/ultrasound needed
mLNP-K30 (F)		1:8	635.95 ± 203.95	0.852 ± 0.148	-1.56 ± 0.87	Slow redispersion. Vortex/ultrasound needed
mLNP-K90 (A)	PVP K90	1:0.5	*null*	*null*	*null*	Not redispersable
mLNP- K90 (B)		1:1	*null*	*null*	*null*	Not redispersable
mLNP- K90 (C)		1:1.5	*null*	*null*	*null*	Not redispersable
mLNP- K90 (D)		1:3	*null*	*null*	*null*	Not redispersable
mLNP- K90 (E)		1:5	*null*	*null*	*null*	Not redispersable
mLNP- K90 (F)		1:8	*null*	*null*	*null*	Not redispersable
mLNP-P6K (A)	PEG6K	1:0.5	*null*	*null*	*null*	Not redispersable
mLNP-P6K (B)		1:1	*null*	*null*	*null*	Not redispersable
mLNP-P6K (C)		1:1.5	*null*	*null*	*null*	Not redispersable
mLNP-P6K (D)		1:3	409.65 ± 70.75	0.782 ± 0.011	-26.25 ± 0.75	Slow redispersion. Vortex/ultrasound needed
mLNP-P6K (E)		1:5	392.76 ± 85.68	0.517 ± 0.060	-18.89 ± 3.77	Slow redispersion. Vortex/ultrasound needed

mLNP-P6K (F)		1:8	475.43 ± 69.67	0.573 ± 0.042	-19.76 ± 3.53	Fast and easy redispersion
mLNP-P10K (A)	PEG10K	1:0.5	*null*	*null*	*null*	Not redispersable
mLNP-P10K (B)		1:1	*null*	*null*	*null*	Not redispersable
mLNP-P10K (C)		1:1.5	*null*	*null*	*null*	Not redispersable
mLNP-P10K (D)		1:3	376.73 ± 91.22	0.518 ± 0.111	-19.33 ± 4.40	Slow redispersion. Vortex/ultrasound needed
mLNP-P10K (E)		1:5	255.22 ± 31.47	0.618 ± 0.096	-18.77 ± 3.06	Slow redispersion. Vortex/ultrasound needed
mLNP-P10K (F)		1:8	285.69 ± 28.13	0.582 ± 0.067	-18.93 ± 3.05	Fast and easy redispersion

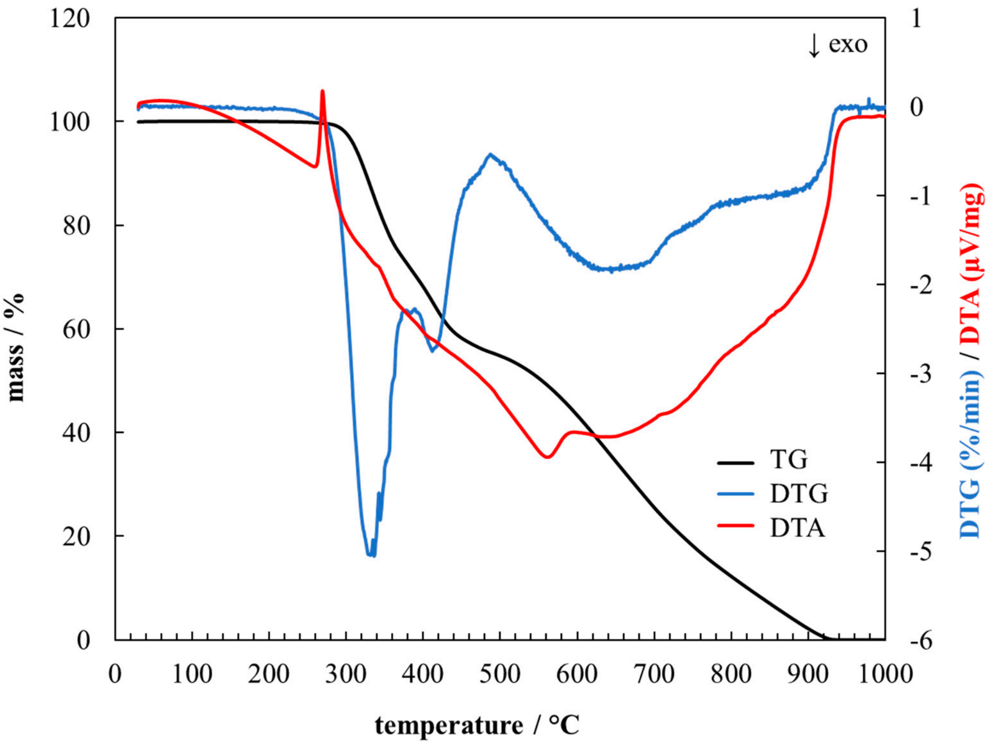

Figure S1. Simultaneous TG/DTA curves for pure RSV.

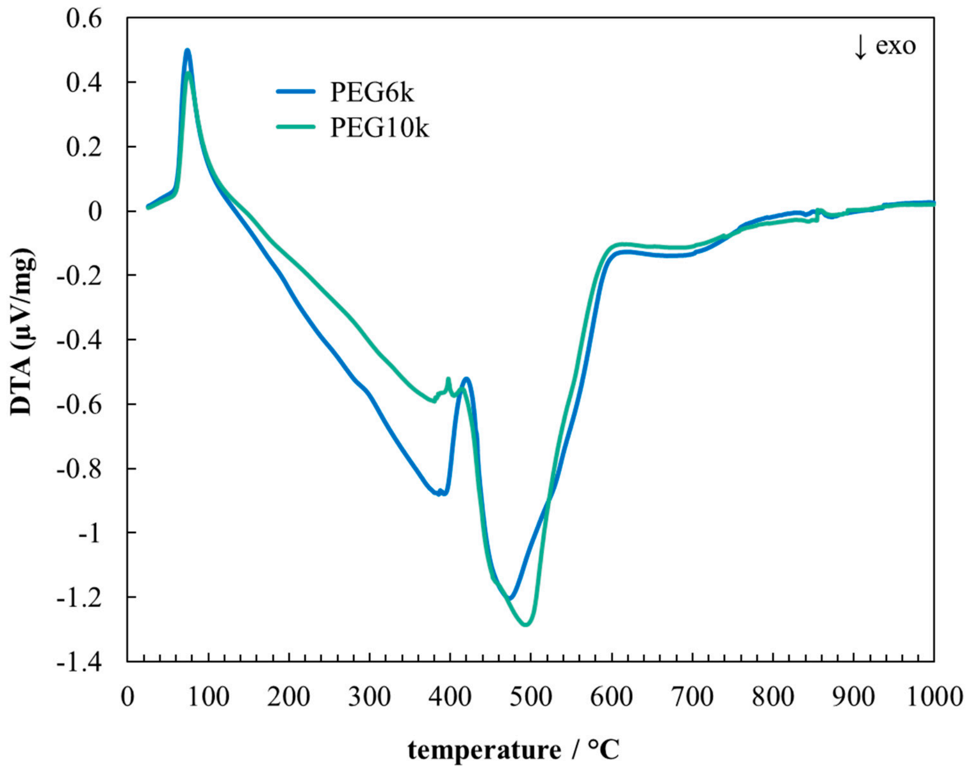

Figure S2. DTA curves for PEG6K and PEG10K.

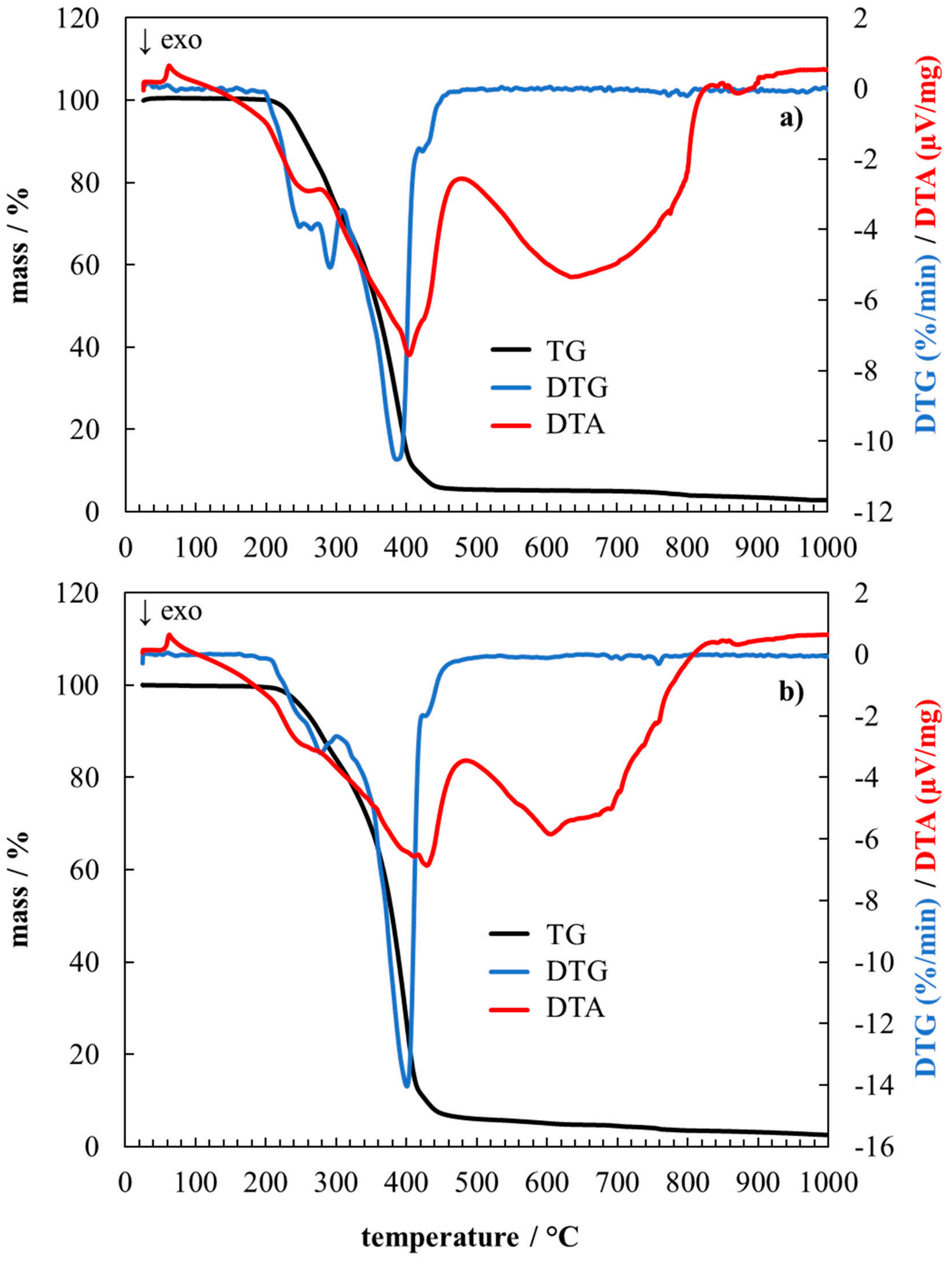

Figure S3. Simultaneous TG/DTA curves for a) mLNP-RSV-P6K and b) mLNP-RSV-P10K.
